# Efficient quantum walk on a quantum processor

**DOI:** 10.1038/ncomms11511

**Published:** 2016-05-05

**Authors:** Xiaogang Qiang, Thomas Loke, Ashley Montanaro, Kanin Aungskunsiri, Xiaoqi Zhou, Jeremy L. O'Brien, Jingbo B. Wang, Jonathan C. F. Matthews

**Affiliations:** 1 Centre for Quantum Photonics, H.H. Wills Physics Laboratory and Department of Electrical and Electronic Engineering, University of Bristol, Bristol BS8 1UB, UK; 2 School of Physics, The University of Western Australia, Crawley, WA 6009, Australia; 3 School of Mathematics, University of Bristol, Bristol BS8 1TW, UK; 4 State Key Laboratory of Optoelectronic Materials and Technologies and School of Physics, Sun Yat-sen University, Guangzhou 510275, China

## Abstract

The random walk formalism is used across a wide range of applications, from modelling share prices to predicting population genetics. Likewise, quantum walks have shown much potential as a framework for developing new quantum algorithms. Here we present explicit efficient quantum circuits for implementing continuous-time quantum walks on the circulant class of graphs. These circuits allow us to sample from the output probability distributions of quantum walks on circulant graphs efficiently. We also show that solving the same sampling problem for arbitrary circulant quantum circuits is intractable for a classical computer, assuming conjectures from computational complexity theory. This is a new link between continuous-time quantum walks and computational complexity theory and it indicates a family of tasks that could ultimately demonstrate quantum supremacy over classical computers. As a proof of principle, we experimentally implement the proposed quantum circuit on an example circulant graph using a two-qubit photonics quantum processor.

## Abstract (web summary)

Quantum walks are a potential framework for developing quantum algorithms, but have so far been limited to analogue quantum-simulation approaches that do not scale. Here, the authors provide a protocol for simulating exponentially large quantum walks using a polynomial number of quantum gates and qubits.

## Graphical abstract

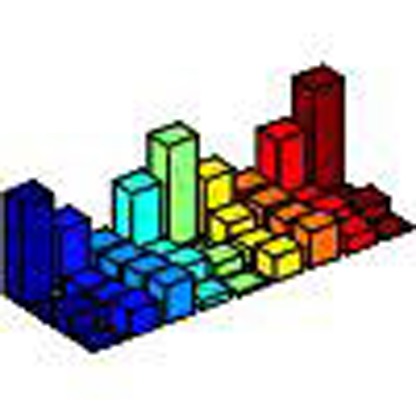

Quantum walks are the quantum mechanical analogue of the well-known classical random walk and they have established roles in quantum information processing^1^,^2^,^3^. In particular, they are central to quantum algorithms created to tackle database search^4^, graph isomorphism^5^,^6^,^7^, network analysis and navigation^8^,^9^, and quantum simulation^10^,^11^,^12^, as well as modelling biological processes^13^,^14^. Meanwhile, physical properties of quantum walks have been demonstrated in a variety of systems, such as nuclear magnetic resonance^15^,^16^, bulk^17^ and fibre^18^ optics, trapped ions^19^,^20^,^21^, trapped neutral atoms^22^ and photonics^23^,^24^. Almost all physical implementations of quantum walk so far followed an analogue approach as for quantum simulation^25^, whereby the apparatus is dedicated to implement specific instances of Hamiltonians without translation onto quantum logic. However, there is no existing method to implement analogue quantum simulations with error correction or fault tolerance, and they do not scale efficiently in resources when simulating broad classes of large graphs. Some exceptions of demonstrations of quantum walks, such as ref. 15, adopted the qubit model, but did not discuss potentially efficient implementation of quantum walks.

Efficient quantum circuit implementations of continuous-time quantum walks (CTQWs) have been presented for sparse and efficiently row-computable graphs^26^,^27^, and specific non-sparse graphs^28^,^29^. However, the design of quantum circuits for implementing CTQWs is in general difficult, since the time-evolution operator is time dependent and non-local^1^. A subset of circulant graphs have the property that their eigenvalues and eigenvectors can be classically computed efficiently^30^,^31^. This enables construction of a scheme that efficiently outputs the quantum state 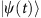, which corresponds to the time-evolution state of a CTQW on corresponding graphs. One can then either implement further quantum circuit operations or perform direct measurements on 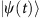 to extract physically meaningful information. For example the ‘SWAP test'^32^ can be used to estimate the similarity of dynamical behaviours of two circulant Hamiltonians operating on two different initial states, as shown in Fig. 1a. This procedure can also be adapted to study the stability of quantum dynamics of circulant molecules (for example, the DNA Möbius strips^33^) in a perturbational environment^34^,^35^. When measuring 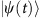 in the computational basis we can sample the probability distribution


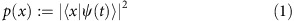


that describes the probability of observing the quantum walker at position *x*∈{0, 1}^*n*^—an *n*-bit string, labelling one of the 2^*n*^ vertices of the given graph, as shown in Fig. 1b. Sampling of this form is sufficient to solve various search and characterization problems^4^,^9^, and can be used to deduce critical parameters of the quantum walk, such as mixing time^2^.

Here we present efficient quantum circuits for implementing CTQWs on circulant graphs with an eigenvalue spectrum that can be classically computed efficiently. These quantum circuits provide the time-evolution states of CTQWs on circulant graphs exponentially faster than best previously known methods^30^. We report a proof-of-principle experiment, where we implement CTQWs on an example circulant graph (namely the complete graph of four vertices) using a two-qubit photonics quantum processor to sample the probability distributions and perform state tomography on the output state of a CTQW. We also provide evidence from computational complexity theory that the probability distributions *p*(*x*) that are output from the circuits of this circulant form are in general hard to sample from using a classical computer, implying our scheme also provides an exponential speedup for sampling. We adapt the methodology of refs 36, 37, 38 to show that if there did exist a classical sampler for a somewhat more general class of circuits, then this would have the following unlikely complexity-theoretic implication: the infinite tower of complexity classes known as the polynomial hierarchy would collapse. This evidence of hardness exists despite the classical efficiency with which properties of the CTQW, such as the eigenvalues of circulant graphs, can be computed on a classical machine.

## Results

### Quantum circuit for CTQW on circulant graph

For an undirected graph *G* of *N* vertices, a quantum particle (or ‘quantum walker') placed on *G* evolves into a superposition 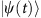 of states in the orthonormal basis 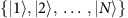 that correspond to vertices of *G*. The exact evolution of the CTQW is governed by connections between the vertices of 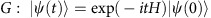 where the Hamiltonian is given by 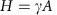 for hopping rate per edge per unit time 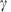 and where *A* is the *N*-by-*N* symmetric adjacency matrix, whose entries are *A*_*jk*_=1, if vertices *j* and *k* are connected by an edge in *G*, and *A*_*jk*_=0 otherwise^1^. The dynamics of a CTQW on a graph with *N* vertices can be evaluated in time poly(*N*) on a classical computer. When a CTQW takes place on a graph *G* of exponential size, that is, *N*=2^*n*^ for an input of size *n*, it becomes interesting to use quantum processors to simulate dynamics.

Circulant graphs are defined by symmetric circulant adjacency matrices for which each row *j* when right rotated by one element, equals the next row *j*+1—for example, complete graphs, cycle graphs and Mobius ladder graphs are all subclasses of circulant graphs, and further examples are shown in Supplementary Note 2. It follows that Hamiltonians for CTQWs on any circulant graph have a symmetric circulant matrix representation, which can be diagonalized by the unitary Fourier transform^31^, that is, *H*=*Q*^†^Λ*Q*, where


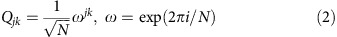


and Λ is a diagonal matrix containing eigenvalues of *H*, which are all real and whose order is determined by the order of the eigenvectors in *Q*. Consequently, we have exp(−*itH*)=*Q*^†^exp(−*it*Λ)*Q*, where the time dependence of exp(−*itH*) is confined to the diagonal unitary operator *D*=exp(−*it*Λ).

The Fourier transformation *Q* can be implemented efficiently by the well-known QFT quantum circuit^39^. For a circulant graph that has *N*=2^*n*^ vertices, the required QFT of *N* dimensions can be implemented with *O*((log*N*)^2^)=*O*(*n*^2^) quantum gates acting on *O*(*n*) qubits. To implement the inverse QFT, the same circuit is used in reverse order with phase gates of opposite sign. *D* can in general be implemented using at most *N*=2^*n*^ controlled-phase gates with phase values being a linear function of *t*, because an arbitrary phase can be applied to an arbitrary basis state, conditional on at most *n*–1 qubits. However, given a circulant graph that has *O*(poly(*n*)) non-zero eigenvalues, only *O*(poly(*n*)) controlled-phase gates are needed to implement *D*. If the given circulant graph has *O*(2^*n*^) distinct eigenvalues, which can be characterized efficiently (such as the cycle graphs and Mobius ladder graphs), then we are still able to implement the diagonal unitary operator *D* using polynomial quantum resources. A general construction of efficient quantum circuits for *D* was given by Childs^40^, and is shown in Supplementary Fig. 1 and Supplementary Note 3 for completeness. Thus, the quantum circuit implementations of CTQWs on circulant graphs can be constructed, which have an overall complexity of *O*(poly(*n*)), and act on at most *O*(*n*) qubits. Compared with the best-known classical algorithm based on fast Fourier transform, that has the computational complexity of *O*(*n*2^*n*^) (ref. 30), the proposed quantum circuit implementation generates the evolution state 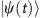 with an exponential advantage in speed.

### Experimental demonstration

To demonstrate implementation of our scheme with two qubits, we have built photonic quantum logic to simulate CTQWs on the *K*_4_ graph—a complete graph with self loops on four vertices (Fig. 2a). The family of complete graphs *K*_*N*_ are a special kind of circulant graph, with an adjacency matrix *A* where *A*_*jk*_=1 for all *j*, *k*. Their Hamiltonian has only 2 distinct eigenvalues, 0 and 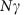. Therefore, the diagonal matrix of eigenvalues of *K*_4_ is 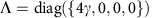. We can readily construct the quantum circuit for implementing CTQWs on *K*_4_ based on diagonalization, using the QFT matrix. However, the choice of using the QFT matrix as the eigenbasis of Hamiltonian is not strictly necessary—any equivalent eigenbasis can be selected. Through the diagonalization using Hadamard eigenbasis, an alternative efficient quantum circuit for implementing CTQWs on *K*_4_ is shown in Fig. 2b, which can be easily extended to *K*_*N*_.

We built a configurable two-qubit photonics quantum processor (Fig. 2c), adapting the entanglement-based technique presented in ref. 41, and implemented CTQWs on *K*_4_ graph with various evolving times and initial states. Specifically, we prepared two different initial states 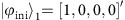 and 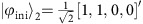, which represent the quantum walker starting from vertex 1, and the superposition of vertices 1 and 2, respectively. We chose the evolution time following the list 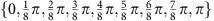, which covers the whole periodical characteristics of CTQWs on *K*_4_ graph. For each evolution, we sampled the corresponding probability distribution with fixed integration time, shown in Fig. 3a,b. To measure how close the experimental and ideal probability distributions are, we calculated the average fidelities defined as 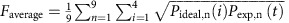. The achieved average fidelities for the samplings with two distinct initial states are 96.68±0.27% and 95.82±0.25%, respectively. Through the proposed circuit implementation, we are also able to examine the evolution states using quantum state tomography, which is generally difficult for the analogue simulations. For two specific evolution states 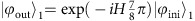 and 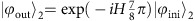, we performed quantum state tomography and reconstructed the density matrices using the maximum likelihood estimation technique. The two reconstructed density matrices achieve fidelities of 85.81±1.08% and 88.44±0.97%, respectively, shown in Fig. 3c,d.

**Figure 1:**
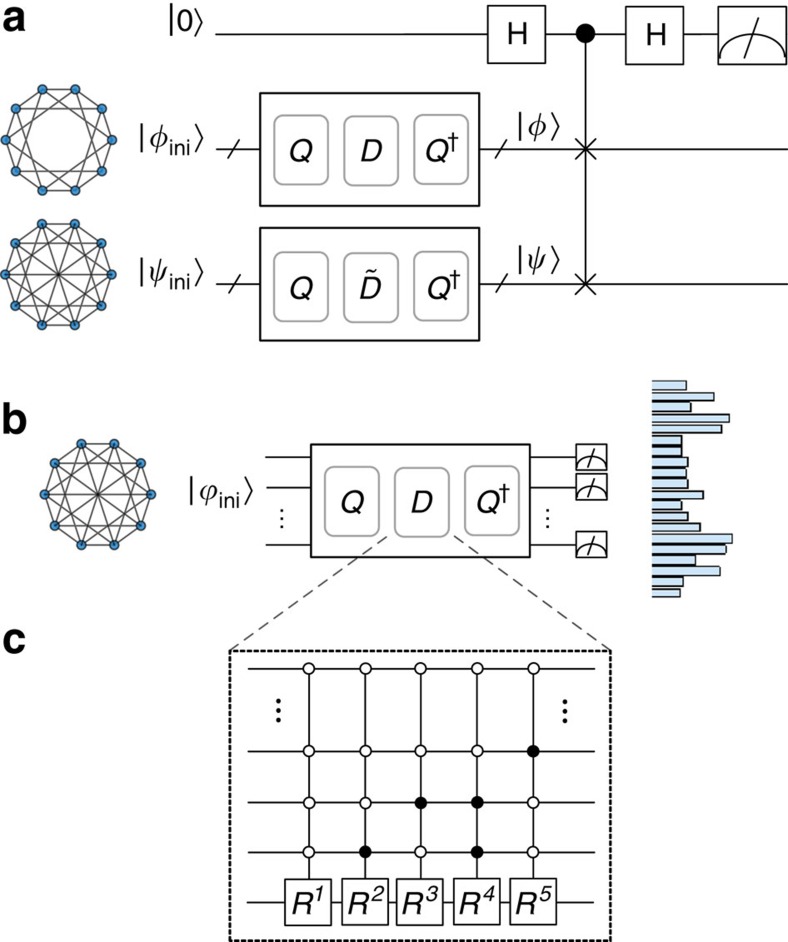
Applications for generating the time-evolution state of circulant Hamiltonians. (**a**) The SWAP test^32^ can be used to estimate the similarity of two evolution states of two similar circulant systems, or when one of the Hamiltonians is non-circulant but efficiently implementable. In brief, an ancillary qubit is entangled with the output states 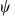 and 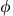 of two compared processes according to 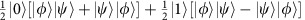. On measuring the ancillary qubit we obtain outcome ‘1' with probability 
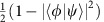
—the probability of observing ‘1' indicates the similarity of dynamical behaviours of the two processes. See its complexity analysis in Supplementary Note 1. (**b**) Probability distributions are sampled by measuring the evolution state in a complete basis, such as the computational basis. (**c**) An example of the quantum circuit for implementing diagonal unitary operator *D*=exp(−*it*Λ), where the circulant Hamiltonian has 5 non-zero eigenvalues. The open and solid circles represent the control qubits as ‘if 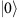
' and ‘if 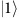
', respectively. 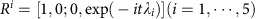, where 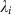 is the corresponding eigenvalue.

Here we have chosen to use *K*_4_ in our experiment because it is simple enough to be implementable with state of the art photonics capability, while it provides an example to demonstrate our protocol for simulating CTQW on a circulant graph with controlled quantum logic. As the size of graph increases, the simplicity of *K*_*N*_ implies that CTQWs on this family of graphs can easily be simulated classically for arbitrary *N*—for CTQW on a complete graph of size *N*, an arbitrary output probability amplitude 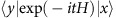 can be readily obtained as 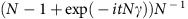 if *x*=*y*, and 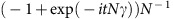 otherwise, where 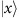 and 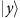 represent the initial state and evolution state, respectively. However, our outlined quantum circuit implementation (Fig. 1) extends to implement CTQW on far more complicated circulant graphs.

### Hardness of the sampling problem

To provide evidence that simulating CTQW on general circulant graphs is likely to be hard classically, we consider a circuit of the form *Q*^†^*DQ*, where *D* is a diagonal matrix made up of poly(*n*) controlled-phase gates and *Q* is the quantum Fourier transform. Define *p*_D_ to be the probability of measuring all qubits to be 0 in the computational basis after *Q*^†^*DQ* is applied to the input state 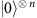. It is readily shown that


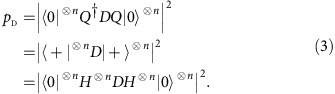


This implies that *p*_D_ can also be obtained through a circuit of form *H*^⊗*n*^*DH*^⊗*n*^ with *D* unchanged—this represents a class of circuits known as instantaneous quantum polynomial time (IQP), which has the following structure: each qubit line begins and ends with a Hadamard (*H*) gate, and, in between, every gate is diagonal in the computational basis^37^,^42^. As such, *p*_D_ is a probability that is classically hard to compute—it is known that computing *p*_D_ for arbitrary diagonal unitaries *D* made up of circuits of poly(*n*) gates, even if each acts on *O*(1) qubits, is #P-hard^38^,^43^,^44^. This hardness result even holds for approximating *p*_D_ up to any relative error strictly less than 1/2 (refs 38, 43, 44), where 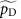 is said to approximate *p*_D_ up to relative error ɛ if


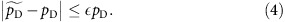


Note that other output probabilities *p*(*x*) cannot be achieved using IQP circuits since a general circulant graph cannot be diagonalized by Hadamard matrices but rather by more heterogeneous Fourier matrices.

Towards a contradiction, assume that there exists a polynomial-time randomized classical algorithm, which samples from *p*, as defined in equation (1). Then a classic result of Stockmeyer^45^ states that there is an algorithm in the complexity class FBPP^NP^, which can approximate any desired probability *p*(*x*) to within relative error *O*(1/poly(*n*)). This complexity class FBPP^NP^—described as polynomial-time randomized classical computation equipped with an oracle to solve arbitrary NP problems—sits within the infinite tower of complexity classes known as the polynomial(-time) hierarchy^46^. Combining with the above hardness result of approximating *p*_D_, we find that the assumption implies that an FBPP^NP^ algorithm solves a #P-hard problem, so P^#P^ would be contained within FBPP^NP^, and therefore the polynomial hierarchy would collapse to its third level. This consequence is considered very unlikely in computational complexity theory^46^. A similar methodology has been used to prove the hardness of IQP and boson sampling^36^,^37^,^38^.

We therefore conclude that, in general, a polynomial-time randomized classical sampler from the distribution *p* is unlikely to exist. Further, this even holds for classical algorithms which sample from any distribution 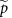 which approximates *p* up to relative error strictly <1/2 in each probability *p*(*x*). It is worth noting that if the output distribution results from measurements on only *O*(poly(log *n*)) qubits^47^, or obeys the sparsity promise that only a poly(*n*)-sized, and *a priori* unknown, subset of the measurement probabilities are non-zero^48^, it could be classically efficiently sampled. It was shown in ref. 38 that assuming certain conjectures in complexity theory, it is classically hard to sample from distributions that are close in total variation distance to arbitrary IQP probability distributions. The differences between circulant and IQP circuits imply that this result does not go through immediately in our setting. Therefore, it remains open to prove hardness of approximate simulation of CTQWs on circulant graphs, which specifically requires to show that computing most of the output probabilities of circulant circuits is hard, assuming some conjectures in complexity theory.

## Discussion

In this paper, we have described how CTQWs on circulant graphs can be efficiently implemented on a quantum computer, if the eigenvalues of the graphs can be characterized efficiently classically. In fact, we can construct an efficient quantum circuit to implement CTQWs on any graph whose adjacency matrix is efficiently diagonalisable, in other words, as long as the matrix of column eigenvectors *Q* and the diagonal matrix of the eigenvalue exponentials *D* can be implemented efficiently. To demonstrate our implementation scheme, we simulated CTQWs on an example 4-vertex circulant graph, *K*_4_, using a two-qubit photonic quantum logic circuit. We have shown that the problem of sampling from the output probability distributions of quantum circuits of the form *Q*^†^*DQ* is hard for classical computers, based on a highly plausible conjecture that the polynomial hierarchy does not collapse. This observation is particularly interesting from both perspectives of CTQW and computational complexity theory, as it provides new insights into the CTQW framework and also helps to classify and identify new problems in computational complexity theory. For the CTQWs on the circulant graphs of poly(*n*) non-zero eigenvalues, the proposed quantum circuit implementations do not need a fully universal quantum computer, and thus can be viewed as an intermediate model of quantum computation. Meanwhile, the evidence we provided for hardness of the sampling problem indicates a promising candidate for experimentally establishing quantum supremacy over classical computers, and further evidence against the extended Church–Turing thesis. To claim in an experiment super-classical performance based on the conjecture outlined in this work, future demonstrations would need to consider circulant graphs that are more general than *K*_*N*_ and that are of sufficient size to be outside the capabilities of a classical computer. For photonics, the biggest challenges remain increasing the number of indistinguishable photons and controlled gate operations. For any platform, quantum circuit implementation of CTQWs could be more appealing due to available methods in fault tolerance and error correction, which are difficult to implement for other intermediate models like boson sampling^49^ and for analogue quantum simulation. Our results may also lead to other practical applications through the use of CTQWs for quantum algorithm design.

## Methods

### Experimental set-up

**Figure 2:**
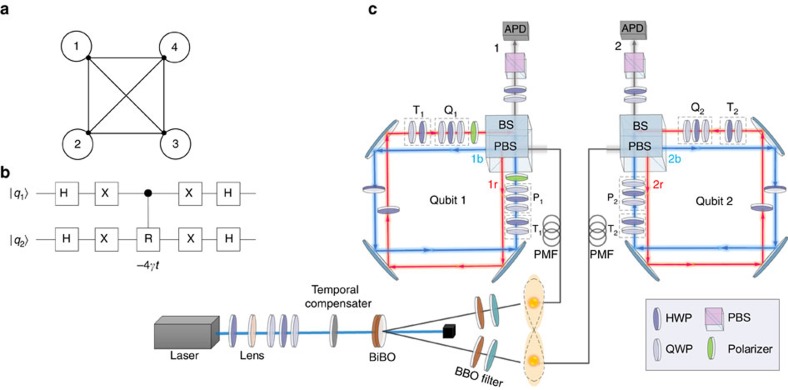
The schematic diagram and set-up of experimental demonstration. (**a**) The *K*_4_ graph. (**b**) The quantum circuit for implementing CTQW on the *K*_4_ graph. This can also be used to implement CTQW on the *K*_4_ graph without self-loops, up to a global phase factor 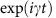. H and X represent the Hadamard and Pauli-X gate, respectively. 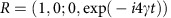 is a phase gate. (**c**) The experimental set-up for a reconfigurable two-qubit photonics quantum processor, consisting of a polarization-entangled photon source using paired type-I BiBO crystal in the sandwich configuration and displaced Sagnac interferometers. See further details in Methods.

**Figure 3:**
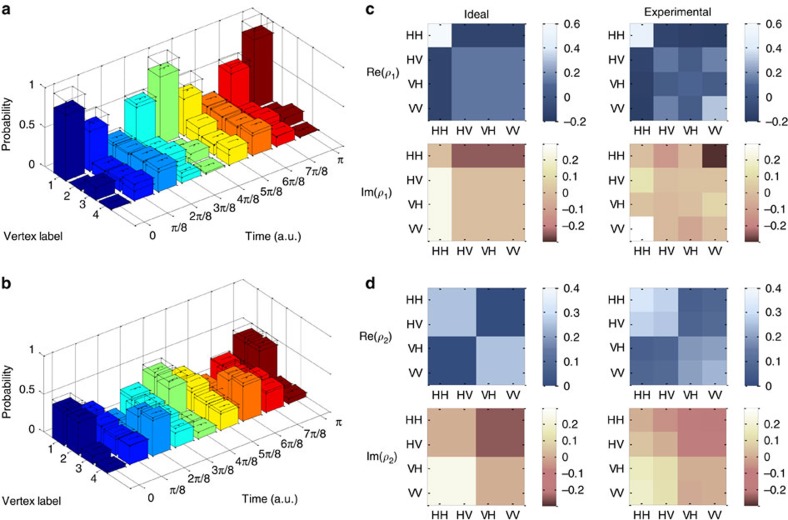
Experimental results for simulating CTQWs on *K*_4_. (**a**,**b**) The experimental sampled probability distributions with ideal theoretical distributions overlaid, for CTQWs on *K*_4_ graph with initial states 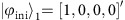 and 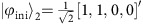. The s.d. of each individual probability is also plotted, which is calculated by propagating error assuming Poissonian statistics. (**c**,**d**) The ideal theoretical and experimentally reconstructed density matrices for the states 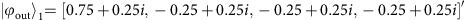 (corresponding to *ρ*_1_) and 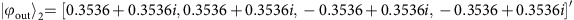 (corresponding to *ρ*_2_). Both of the real and imaginary parts of the density matrices are obtained through the maximum likelihood estimation technique, and is shown as Re(*ρ*) and Im(*ρ*), respectively. Further results are shown in Supplementary Table 1, Supplementary Fig. 2 and Supplementary Note 4.

A diagonally polarized, 120 mW, continuous-wave laser beam with central wavelength of 404 nm is focused at the centre of paired type-I BiBO crystals with their optical axes orthogonally aligned to each other, to create the polarization entangled photon-pairs^50^. Through the spontaneous parametric downconversion process, the photon pairs are generated in the state of 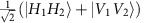, where *H* and *V* represent horizontal and vertical polarization, respectively. The photons pass through the polarization beam-splitter (PBS) part of the dual PBS/beam-splitter cubes on both arms to generate two-photon four-mode state of the form 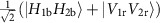 (where r and b labels the red and blue paths shown in Fig. 2c, respectively). Rotations *T*_1_ and *T*_2_ on each path, consisting of half wave-plate (HWP) and quarter wave plate (QWP), convert the state into 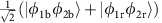, where 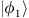 and 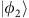 can be arbitrary single-qubit states. The four spatial modes 1b, 2b, 1r and 2r pass through four single-qubit quantum gates *P*_1_, *P*_2_, *Q*_1_ and *Q*_2_, respectively, where each of the four gates is implemented through three wave plates: QWP, HWP and QWP. The spatial modes 1b and 1r (2b and 2r) are then mixed on the beam-splitter part of the cube. By post-selecting the case where the two photons exit at ports 1 and 2, we obtain the state 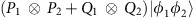. In this way, we implement a two-qubit quantum operation of the form *P*_1_⊗*P*_2_+*Q*_1_⊗*Q*_2_ on the initialized state 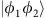.

As shown in Fig. 2b, the quantum circuit for implementing CTQW on the *K*_4_ graph consists of Hadamard gates (H), Pauli-X gates (X) and controlled-phase gate (CP). CP is implemented by configuring 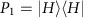, *P*_2_=*I*, 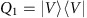, 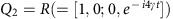, where *P*_1_ and *Q*_1_ are implemented by polarizers. Altogether with combining the operation (H·X)⊗(H·X) before CP with state preparation and the operation (X·H)⊗X·H after CP with measurement setting, we implement the whole-quantum circuit on the experimental set-up. The evolution time of CTQW is controlled by the phase value of *R*, which is determined by setting the three wave plates of *Q*_2_ in Fig. 2c to 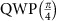, 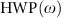, 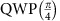, where the angle 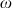 of HWP equals to the phase of *R*:−4γ t. The evolution time *t* is then given by 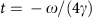.

## Additional information

**How to cite this article:** Qiang, X. *et al*. Efficient quantum walk on a quantum processor. *Nat. Commun.* 7:11511 doi: 10.1038/ncomms11511 (2016).

## Supplementary Material

Supplementary Information Supplementary Figures 1-2, Supplementary Table 1, Supplementary Notes 1-4 and Supplementary References
