# Supplementary material for: Efficient quantum walk on a quantum processor
Source: Nat Commun. 2016 May 5;7:11511. doi: 10.1038/ncomms11511 (PMC4858748; doi:10.1038/ncomms11511)
Supplement: Supplementary Information — Supplementary Figures 1-2, Supplementary Table 1, Supplementary Notes 1-4 and Supplementary References [file ncomms11511-s1.pdf]

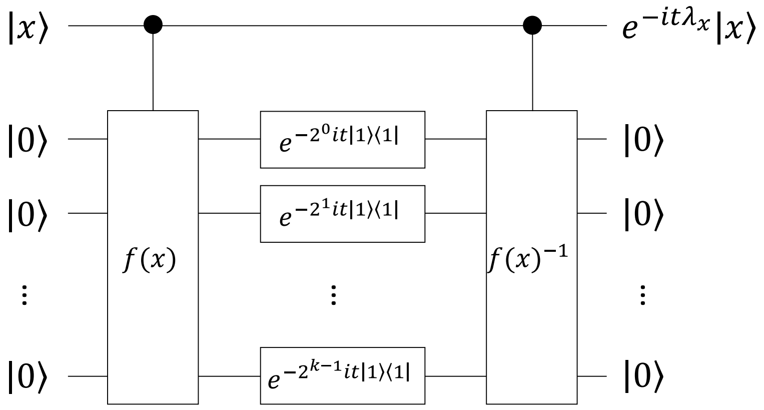

Supplementary Figure 1: **The quantum circuit for implementing the diagonal unitary operator of given circulant Hamiltonian.** The eigenvalues of given circulant can be calculated by the function $f(x)$ efficiently classically.

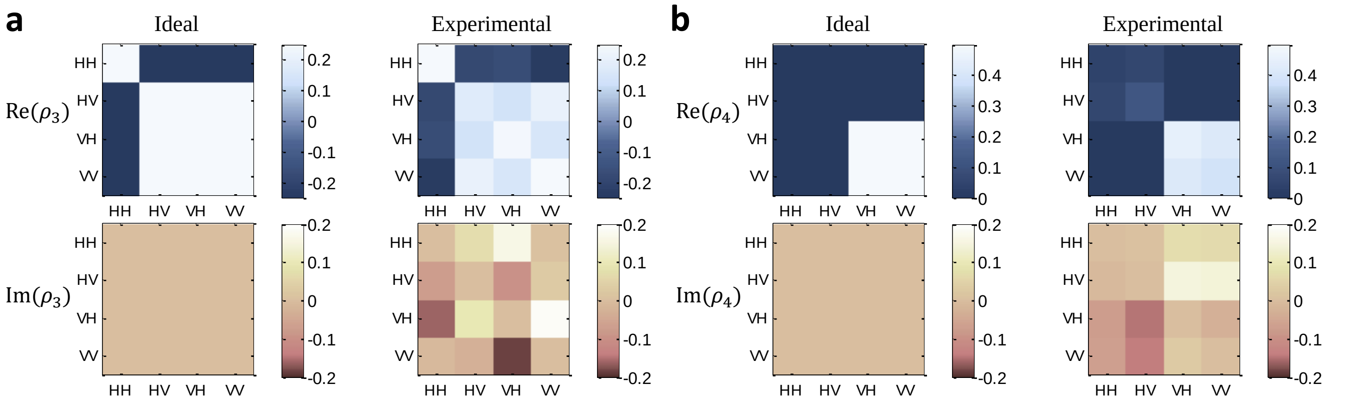

Supplementary Figure 2: **The ideal theoretical and experimentally reconstructed density matrices for the evolution states of CTQWs on K_4.** (**a**) The evolution state $|\varphi_{\text{out}}\rangle_3 = \frac{1}{2}[1, -1, -1, -1]'$ (corresponding to ρ_3). (**b**) The evolution state $|\varphi_{\text{out}}\rangle_4 = \frac{1}{\sqrt{2}}[0, 0, -1, -1]'$ (corresponding to ρ_4). Both of the real and imaginary parts of the density matrices are obtained through the maximum likelihood estimation technique, and shown as Re(ρ) and Im(ρ) respectively. The achieved fidelities are 88.63±1.24% and 91.53±0.43% respectively.

Node/T	0	$\frac{1}{8}\pi$	$\frac{2}{8}\pi$	$\frac{3}{8}\pi$	$\frac{4}{8}\pi$	$\frac{5}{8}\pi$	$\frac{6}{8}\pi$	$\frac{7}{8}\pi$	π
$P_{1,\text{ideal}}$	1	0.625	0.25	0.625	1	0.625	0.25	0.625	1
$P_{2,\text{ideal}}$	0	0.125	0.25	0.125	0	0.125	0.25	0.125	0
$P_{3,\text{ideal}}$	0	0.125	0.25	0.125	0	0.125	0.25	0.125	0
$P_{4,\text{ideal}}$	0	0.125	0.25	0.125	0	0.125	0.25	0.125	0
$P_{1,\text{exp}}$	0.8225	0.5014	0.2139	0.5759	0.8482	0.4583	0.2414	0.549	0.858
$P_{2,\text{exp}}$	0.003	0.1388	0.2254	0.1455	0.0078	0.2167	0.2375	0.1324	0.0114
$P_{3,\text{exp}}$	0.1598	0.153	0.2659	0.2105	0.1284	0.1333	0.2299	0.1912	0.1193
$P_{4,\text{exp}}$	0.0148	0.2068	0.2948	0.0681	0.0156	0.1917	0.2912	0.1275	0.0114

Node/T	0	$\frac{1}{8}\pi$	$\frac{2}{8}\pi$	$\frac{3}{8}\pi$	$\frac{4}{8}\pi$	$\frac{5}{8}\pi$	$\frac{6}{8}\pi$	$\frac{7}{8}\pi$	π
$P_{1,\text{ideal}}$	0.5	0.25	0	0.25	0.5	0.25	0	0.25	0.5
$P_{2,\text{ideal}}$	0.5	0.25	0	0.25	0.5	0.25	0	0.25	0.5
$P_{3,\text{ideal}}$	0	0.25	0.5	0.25	0	0.25	0.5	0.25	0
$P_{4,\text{ideal}}$	0	0.25	0.5	0.25	0	0.25	0.5	0.25	0
$P_{1,\text{exp}}$	0.4386	0.2796	0.0682	0.2927	0.4375	0.2823	0.0669	0.2338	0.434
$P_{2,\text{exp}}$	0.4189	0.2607	0.0746	0.2717	0.4103	0.3008	0.0605	0.2	0.4415
$P_{3,\text{exp}}$	0.1031	0.2156	0.3945	0.2482	0.0679	0.2058	0.4108	0.2923	0.0566
$P_{4,\text{exp}}$	0.0395	0.2441	0.4627	0.1874	0.0842	0.2111	0.4618	0.2738	0.0679

Node/T	0	$\frac{1}{8}\pi$	$\frac{2}{8}\pi$	$\frac{3}{8}\pi$	$\frac{4}{8}\pi$	$\frac{5}{8}\pi$	$\frac{6}{8}\pi$	$\frac{7}{8}\pi$	π
$P_{1,\text{ideal}}$	0.5	0.5	0.5	0.5	0.5	0.5	0.5	0.5	0.5
$P_{2,\text{ideal}}$	0.5	0.5	0.5	0.5	0.5	0.5	0.5	0.5	0.5
$P_{3,\text{ideal}}$	0	0	0	0	0	0	0	0	0
$P_{4,\text{ideal}}$	0	0	0	0	0	0	0	0	0
$P_{1,\text{exp}}$	0.4147	0.3918	0.3865	0.4289	0.4156	0.382	0.4058	0.4356	0.4123
$P_{2,\text{exp}}$	0.4627	0.474	0.4679	0.4348	0.4359	0.4697	0.4507	0.4189	0.4416
$P_{3,\text{exp}}$	0.0601	0.0671	0.0722	0.0782	0.0898	0.0877	0.0792	0.0933	0.0823
$P_{4,\text{exp}}$	0.0625	0.0671	0.0734	0.0581	0.0587	0.0606	0.0642	0.0522	0.0639

Node/T	0	$\frac{1}{8}\pi$	$\frac{2}{8}\pi$	$\frac{3}{8}\pi$	$\frac{4}{8}\pi$	$\frac{5}{8}\pi$	$\frac{6}{8}\pi$	$\frac{7}{8}\pi$	π
$P_{1,\text{ideal}}$	0.5	0.125	0.25	0.625	0.5	0.125	0.25	0.625	0.5
$P_{2,\text{ideal}}$	0.5	0.625	0.25	0.125	0.5	0.625	0.25	0.125	0.5
$P_{3,\text{ideal}}$	0	0.125	0.25	0.125	0	0.125	0.25	0.125	0
$P_{4,\text{ideal}}$	0	0.125	0.25	0.125	0	0.125	0.25	0.125	0
$P_{1,\text{exp}}$	0.4178	0.1655	0.1969	0.4932	0.4729	0.1492	0.1977	0.4217	0.4332
$P_{2,\text{exp}}$	0.3541	0.548	0.2749	0.1504	0.3824	0.6258	0.2503	0.1085	0.4308
$P_{3,\text{exp}}$	0.1376	0.1015	0.211	0.1467	0.0594	0.1047	0.2316	0.2796	0.0734
$P_{4,\text{exp}}$	0.0904	0.185	0.3171	0.2096	0.0853	0.1203	0.3205	0.1902	0.0626

Node/T	0	$\frac{1}{8}\pi$	$\frac{2}{8}\pi$	$\frac{3}{8}\pi$	$\frac{4}{8}\pi$	$\frac{5}{8}\pi$	$\frac{6}{8}\pi$	$\frac{7}{8}\pi$	π
$P_{1,\text{ideal}}$	0.25	0.5	0.25	0	0.25	0.5	0.25	0	0.25
$P_{2,\text{ideal}}$	0.25	0	0.25	0.5	0.25	0	0.25	0.5	0.25
$P_{3,\text{ideal}}$	0.25	0.5	0.25	0	0.25	0.5	0.25	0	0.25
$P_{4,\text{ideal}}$	0.25	0	0.25	0.5	0.25	0	0.25	0.5	0.25
$P_{1,\text{exp}}$	0.2642	0.4163	0.2227	0.0172	0.2191	0.4136	0.2167	0.0591	0.2476
$P_{2,\text{exp}}$	0.2724	0.0156	0.3	0.4678	0.2367	0.0227	0.2808	0.4864	0.199
$P_{3,\text{exp}}$	0.2561	0.5525	0.2409	0.0043	0.3145	0.5545	0.2266	0.0227	0.3204
$P_{4,\text{exp}}$	0.2073	0.0156	0.2364	0.5107	0.2297	0.0091	0.2759	0.4318	0.233

Node/T	0	$\frac{1}{8}\pi$	$\frac{2}{8}\pi$	$\frac{3}{8}\pi$	$\frac{4}{8}\pi$	$\frac{5}{8}\pi$	$\frac{6}{8}\pi$	$\frac{7}{8}\pi$	π
$P_{1,\text{ideal}}$	0.25	0.625	0.5	0.125	0.25	0.625	0.5	0.125	0.25
$P_{2,\text{ideal}}$	0.25	0.125	0	0.125	0.25	0.125	0	0.125	0.25
$P_{3,\text{ideal}}$	0.25	0.125	0	0.125	0.25	0.125	0	0.125	0.25
$P_{4,\text{ideal}}$	0.25	0.125	0.5	0.625	0.25	0.125	0.5	0.625	0.25
$P_{1,\text{exp}}$	0.2056	0.4226	0.3307	0.1129	0.168	0.4883	0.3425	0.1321	0.3347
$P_{2,\text{exp}}$	0.1542	0.2469	0.0906	0.0887	0.127	0.1596	0.0827	0.0566	0.3431
$P_{3,\text{exp}}$	0.2477	0.1674	0.0354	0.1411	0.3238	0.1643	0.0276	0.1358	0.1715
$P_{4,\text{exp}}$	0.3925	0.1632	0.5433	0.6573	0.3811	0.1878	0.5472	0.6755	0.1506

Supplementary Table 1: The ideal theoretical and experimental probability distributions of CTQWs on K_4 graph with various initial states. The six sub-tables from the top down correspond to CTQWs with initial states $|\varphi_{\text{ini}}\rangle_1 = [1,0,0,0]'$, $|\varphi_{\text{ini}}\rangle_2 = \frac{1}{\sqrt{2}}[1,1,0,0]'$, $|\varphi_{\text{ini}}\rangle_3 = \frac{1}{\sqrt{2}}[1,-1,0,0]'$, $|\varphi_{\text{ini}}\rangle_4 = \frac{1}{\sqrt{2}}[1,-i,0,0]'$, $|\varphi_{\text{ini}}\rangle_5 = \frac{1}{2}[1,i,1,i]'$ and $|\varphi_{\text{ini}}\rangle_6 = \frac{1}{2}[1,i,i,-1]'$ respectively. In each sub-table, the first four rows show ideal results and the second four rows show experimental results.

Supplementary Note 1 Complexity analysis of "swap test"

Unlike the sampling problem we discussed in the main text, the scenario of "SWAP test", where we compare two unitary processes $Q^\dagger DQ$ and $Q^\dagger \widetilde{D}Q$, could sometimes be easier for a classical computer. Imagine we start each process in the state $|\phi_{\text{ini}}\rangle = |\psi_{\text{ini}}\rangle = |0\rangle^{\otimes n}$. Then the overlap O between the resulting output states approximated by the SWAP test satisfies

$$\begin{aligned} O &= |\,\langle 0|^{\otimes n}\,(Q^\dagger DQ)(Q^\dagger \widetilde{D}Q)\,|0\rangle^{\otimes n}\,|^2 \\ &= |\,\langle +|^{\otimes n}\,D\widetilde{D}\,|+\rangle^{\otimes n}\,|^2 = \Big|\frac{1}{2^n}\sum_{x\in\{0,1\}^n} D_{xx}\widetilde{D}_{xx}\Big|^2, \end{aligned} \tag{1}$$

where D_{xx} is the value at position x on the diagonal of D. O can be approximated by a classical algorithm up to $O(1/\operatorname{poly}(n))$ additive error. The algorithm simply takes the average of $\operatorname{poly}(n)$ values of the product $D_{xx}\widetilde{D}_{xx}$ for uniformly random x. For each x, this value can be computed exactly in polynomial time.

This highlights that the complexity of comparing $Q^\dagger DQ\,|\phi_{\text{ini}}\rangle$ and $Q^\dagger \widetilde{D}Q\,|\psi_{\text{ini}}\rangle$ depends on the choice of input states $|\phi_{\text{ini}}\rangle$ and $|\psi_{\text{ini}}\rangle$. In full generality, one could allow these to be arbitrary states produced by a polynomial-time quantum computation; the state comparison problem would then be BQP-complete, but for rather trivial reasons. We expect that the problem would remain classically hard for choices of initial states relevant, for example, to quantum-chemistry applications. On the other hand, the SWAP test can still be used as in the main text to compare the evolution of two Hamiltonians, one of which is not circulant but is efficiently implementable. In this case, the comparison problem is also BQP-complete, and hence expected to be hard for a classical computer.

Supplementary Note 2 Further details on circulant graphs and other examples

A circulant graph of N vertices is fully described by an N-by-N symmetric circulant adjacency matrix C defined as follows.

$$C = \begin{bmatrix} c_0 & c_1 & c_2 & \cdots & c_{N-1} \\ c_{N-1} & c_0 & c_1 & \cdots & c_{N-2} \\ c_{N-2} & c_{N-1} & c_0 & \cdots & c_{N-3} \\ \vdots & \vdots & \vdots & \ddots & \vdots \\ c_1 & c_2 & c_3 & \cdots & c_0 \end{bmatrix} \tag{2}$$

where $c_j = c_{N-j}, j = 1, 2, \cdots, N-1$. Obviously, every circulant matrix can be generated given any row of the matrix – conventionally we use the first row of the matrix, denoted as r_C. It is clear that C has at most N distinct eigenvalues which are given by $\lambda_m = \sum_{k=0}^{N-1} c_k \omega^{-mk}$, where $\omega = \exp(2\pi i/N)$ and $m = 0, 1, \ldots, N-1$ [1]. If C is singular, some of the eigenvalues of C are zeros. The complete graph and complete bipartite graph are straightforward examples of circulant graphs with few distinct eigenvalues.

There are also some other interesting examples of circulant graph such as self-complementary circulant graphs and Paley graphs with prime order [2, 3]. Both of these two families of graphs are also strongly regular graphs which have only three distinct eigenvalues. For example, the Paley graph on 13 vertices has three distinct eigenvalues: 6 (with multiplicity 1) and $\frac{1}{2}\left(-1 \pm \sqrt{13}\right)$ (both with multiplicity 6), and thus the diagonal unitary $\exp(-it\Lambda)$ can be implemented efficiently. We note here it is required to implement QFT (and its inverse) for the dimension of 13, which does not have the form of $N = 2^n$. The QFT on general dimensions can be implemented by means of amplitude amplification with extra qubit registers to perform the computation [4]. Alternatively, approximate versions of the QFT on general dimensions have also been developed [5].

Supplementary Note 3 Implementation of the diagonal unitary operator

We say that the eigenvalues of a circulant graph can be characterised efficiently, if they can be calculated efficiently classically. In other words, the eigenvalue matrix Λ of the given circulant Hamiltonian can be efficiently computed, and thus the diagonal unitary operator $\exp(-it\Lambda)$ can be efficiently implemented [6]. Specifically, there exists a quantum circuit shown in Supplementary Figure 1, which transforms a computational basis state $|x\rangle$, together with a k-qubit

ancilla $|0\rangle$ for $k = \text{poly}(n)$, as

$$\begin{aligned} |x\rangle\,|0\rangle &\rightarrow |x\rangle\,|\lambda_x\rangle \\ &\rightarrow e^{-it\lambda_x}\,|x\rangle\,|\lambda_x\rangle \\ &\rightarrow e^{-it\lambda_x}\,|x\rangle\,|0\rangle = e^{-it\Lambda}\,|x\rangle\,|0\rangle \end{aligned} \tag{3}$$

where $x = 0, 1, \ldots, N-1$. Note that here we assume λ_x can be expressed exactly as a rational number with k bits of precision. If this is not the case, truncating λ_x to k bits of precision will introduce an error which can be made arbitrarily small by taking large enough $k = \text{poly}(n)$. The function $f(x)$ returns λ_x for any given x. λ_x is always a real number since the adjacency matrix is symmetric.

For example, for the case of the cycle graph of $N = 2^n$ vertices, there are essentially $N/2$ distinct eigenvalues simply given by $\lambda_x = 2\cos(2\pi x/N)$, where $x = 0, 1, \ldots, N-1$. And then $f(x)$ will be the cosine function that can be computed with a number of operations polynomial in n, using a reversible equivalent of classical algorithms to compute trigonometric functions, e.g. the Taylor approximation. In general, given a sparse circulant graph which has only poly(n) 1s in the first row r_C of its adjacency matrix, an efficient function $f(x)$ can be given as

$$f(x) = \sum_{y \in S} e^{2i\pi xy/N} \tag{4}$$

where S is the set of positions for which the first row in nonzero. $f(x)$ is a sum of $|S| = \text{poly}(n)$ numbers, taking $O(\text{poly}(n))$ time to compute. For a non-sparse circulant graph, its eigenvalues are still possible to be calculated efficiently classically. Some straightforward examples are complete graph, complete bipartite graph $K_{N,N}$ and cocktail party graph. Therefore, together with the quantum circuits of QFT and the inverse of QFT, we construct an efficient quantum circuit for implementing CTQW on the circulant graph whose eigenvalues can be computed efficiently classically.

Supplementary Note 4 Further experimental results

We present the ideal and experimentally sampled probability distributions of CTQW with initial states $|\varphi_{\text{ini}}\rangle_1 = [1, 0, 0, 0]'$, $|\varphi_{\text{ini}}\rangle_2 = \frac{1}{\sqrt{2}}[1, 1, 0, 0]'$ (mentioned in the main text), $|\varphi_{\text{ini}}\rangle_3 = \frac{1}{\sqrt{2}}[1, -1, 0, 0]'$, $|\varphi_{\text{ini}}\rangle_4 = \frac{1}{\sqrt{2}}[1, -i, 0, 0]'$, $|\varphi_{\text{ini}}\rangle_5 = \frac{1}{2}[1, i, 1, i]'$, $|\varphi_{\text{ini}}\rangle_6 = \frac{1}{2}[1, i, i, -1]'$, in Supplementary Table 1. The achieved average fidelities between ideal and experimental probability distributions are 96.68±0.27%, 95.82±0.25%, 92.61±0.21%, 96.36±0.16%, 98.76±0.17% and 97.27±0.24% respectively. In the main text, we reconstructed the density matrices for the two quantum states $|\varphi_{\text{out}}\rangle_1$ and $|\varphi_{\text{out}}\rangle_2$, through performing quantum state tomography. Here we also present the reconstructed density matrices for another two evolution states $|\varphi_{\text{out}}\rangle_3 = \exp(-iH\frac{3}{4}\pi)|\varphi_{\text{ini}}\rangle_1$ and $|\varphi_{\text{out}}\rangle_4 = \exp(-iH\frac{3}{4}\pi)|\varphi_{\text{ini}}\rangle_2$, with the achieved fidelities of 88.63±1.24% and 91.53±0.53% respectively. See in Supplementary Figure 2. The four reconstructed density matrices ρ_1, ρ_2, ρ_3 and ρ_4 for quantum states $|\varphi_{\text{out}}\rangle_1$, $|\varphi_{\text{out}}\rangle_2$, $|\varphi_{\text{out}}\rangle_3$ and $|\varphi_{\text{out}}\rangle_4$ are shown as follows.

$$\rho_1 = \begin{bmatrix} 0.4763 & -0.1175 - 0.1281i & -0.1410 - 0.0112i & -0.1507 - 0.3104i \\ -0.1175 + 0.1281i & 0.1354 & 0.0257 + 0.0115i & 0.1620 + 0.0133i \\ -0.1410 + 0.0112i & 0.0257 - 0.0115i & 0.0841 & 0.0289 + 0.0797i \\ -0.1507 + 0.3104i & 0.1620 - 0.0133i & 0.0289 - 0.0797i & 0.3041 \end{bmatrix} \tag{5}$$

$$\rho_2 = \begin{bmatrix} 0.3207 & 0.2801 - 0.0479i & 0.0665 - 0.1666i & 0.0851 - 0.1810i \\ 0.2801 + 0.0479i & 0.2575 & 0.0812 - 0.1135i & 0.0989 - 0.1245i \\ 0.0665 + 0.1666i & 0.0812 + 0.1135i & 0.1899 & 0.2044 + 0.0088i \\ 0.0851 + 0.1840i & 0.0989 + 0.1245i & 0.2044 - 0.0088i & 0.2319 \end{bmatrix} \tag{6}$$

$$\rho_3 = \begin{bmatrix} 0.2779 & -0.1804 + 0.0749i & -0.1711 + 0.1613i & -0.2397 + 0.0091i \\ -0.1804 - 0.0749i & 0.1778 & 0.1376 - 0.0938i & 0.2083 + 0.0311i \\ -0.1711 - 0.1613i & 0.1376 + 0.0938i & 0.2411 & 0.1507 + 0.1825i \\ -0.2397 - 0.0091i & 0.2083 - 0.0311i & 0.1507 - 0.1825i & 0.3031 \end{bmatrix} \tag{7}$$

$$\rho_4 = \begin{bmatrix} 0.0418 & 0.0605+0.0067i & -0.0353+0.0698i & -0.0287+0.0671i \\ 0.0605-0.0067i & 0.1196 & -0.0260+0.1465i & -0.0155+0.1381i \\ -0.0353-0.0698i & -0.0260-0.1465i & 0.4481 & 0.4175-0.0263i \\ -0.0287-0.0671i & -0.0155-0.1381i & 0.4175+0.0263i & 0.3904 \end{bmatrix} \tag{8}$$

Supplementary References

[1] Ng, M. K. *Iterative Methods for Toeplitz Systems.* Oxford University Press New York (2004).

[2] Zhou, H. On self-complementary of circulant graphs. In *Theoretical and Mathematical Foundations of Computer Science* 464-471, Springer (2011).

[3] Rajasingh, I. & Natarajan, P. Spanning trees in circulant networks. In *The 6th International Conference on Information Technology* (2013).

[4] Mosca, M. & Zalka, C. Exact quantum fourier transforms and discrete logarithm algorithms. *Int. J. Quantum Inf.* **2** 91-100 (2004).

[5] Hales, L. & Hallgren, S. An improved quantum fourier transform algorithm and applications. In *Foundations of Computer Science, 2000. Proceedings. 41st Annual Symposium on* 515-525, IEEE (2000).

[6] Childs, A. M. *Quantum information processing in continuous time.* PhD Thesis, Massachusetts Institute of Technology (2004).
